# Machine Learning-Based Classification for Crop-Type Mapping Using the Fusion of High-Resolution Satellite Imagery in a Semiarid Area

**DOI:** 10.1155/2021/8810279

**Published:** 2021-04-20

**Authors:** Aicha Moumni, Abderrahman Lahrouni

**Affiliations:** Faculty of Science Semlalia, Cadi Ayyad University, Marrakesh 40000, Morocco

## Abstract

The monitoring of cultivated crops and the types of different land covers is a relevant environmental and economic issue for agricultural lands management and crop yield prediction. In this context, this paper aims to use and evaluate the contribution of multisensors classification based on machine learning classifiers to crop-type identification in a semiarid area of Morocco. It is a very heterogeneous zone characterized by mixed crops (tree crops with annual crops, same crop with different phenological states during the same agricultural season, crop rotation, etc.). Therefore, such heterogeneity made the crop-type discrimination more complicated. To overcome these challenges, the present work is the first study in this area which used the fusion of high spatiotemporal resolution Sentinel-1 and Sentinel-2 satellite images for land use and land cover mapping. Three machine learning classifier algorithms, artificial neural network (ANN), support vector machine (SVM), and maximum likelihood (ML), were applied to identify and map crop types in irrigated perimeter. In situ observations of the year 2018, for the R3 perimeter of Haouz plain in central Morocco, were used with satellite data of the same year to perform this work. The results showed that combined images acquired in C-band and the optical range improved clearly the crop-type classification performance (overall accuracy = 89%; Kappa = 0.85) compared to the classification results of optical or SAR data alone.

## 1. Introduction

Accurate and detailed knowledge of land cover/land use (LC/LU) is a crucial issue for research work and many operational applications in agriculture such as a crop water requirement, crop yield prediction, etc. The availability of remote sensing imagery, that offered the access to a set of large regions, is a major asset to elaborate LC/LU maps. Remote sensors operate on a variety of basic physical principles, recording the electromagnetic properties of an Earth's surface (i.e., the energy reflected (optical sensors), emitted (passive infrared or microwave thermal sensors), or diffused (active radar sensors)) and, therefore, provide a variety of information on the properties of land cover [1]. The use of optical remote sensing for LC/LU mapping is well established and can be considered effective, yet it exhibits some shortcomings when applied to large scale regions with complex land cover, or where cloud cover is frequent [2–5]. On the other hand, using SAR data for crop-type discrimination is still facing many challenges, particularly that its recognition accuracy is not high enough [6, 7]. But it has been shown that it can provide improvements in classification quality when combined with optical data [8–10]. In order to achieve these improvements and better identify land cover types, combining datasets acquired from remote sensors that rely on different physical fundamentals, and thus providing synergistic information on surface properties, leads to a promising approach [11–13], particularly with the recent free of charge image datasets (optical and radar images from the Sentinel satellite sensors) [14], which provide the possibility of data fusion of higher spectral resolution, compensating the limits of the use of unique data products alone. Nowadays, studies support the hypothesis that the complementarity of these two types of data is able to provide improved information on LC/LU applications. For example, assume that the optical energy reflected by vegetation depends on the structure of the leaf, pigmentation, and humidity, while the microwave energy dispersed by vegetation depends on the size, the density, the orientation, and the dielectric properties of elements comparable to the wavelength size of the radar [15]. On the basis of this hypothesis, the objective of this study was to evaluate the usefulness of using SAR data on the one hand and of the combination of both types of remote sensing data, on the other hand, to map and identify crop types.

The work begins with the description of test site and the satellite data acquired and detailed the methodology to be followed. 20 Sentinel-2 (S2) optical images and 12 Sentinel-1 (S1) SAR images have been downloaded. In addition, in situ dataset was acquired from a field and supplemented with data from Google Earth platform. The methodology consists of the application of the ML, SVM, and ANN machine learning algorithms for the classification of Sentinel-2 products, Sentinel-1 products and the combined products of the two sensors. The quantitative evaluation of the results was carried out by the overall accuracy (OA) and the Kappa coefficient (K) obtained by construction of the confusion matrix of each classified image, while the qualities of the images were compared visually.

Moreover, these results were followed by comparison between the performance of classification using optical imagery, SAR imagery, and the combination of both of datasets and discussion in order to evaluate the performance of using radar data for the production of LC/LU maps of reliable soil, and prove the possibility of improving classification accuracy by synergistic use and the fusion of data acquired from multisensors. Finally, the paper ended with the main conclusions and perspectives extracted from this study.

## 2. Materials and Methods

### 2.1. Test Site

The plain of Haouz is a vast plain of 6000 km^2^ of surface which stretches over a length of approximately 150 km from east to west in the region of Marrakech-Safi located in central Morocco and including part of the Haut-Atlas. It is a predominantly rural area where the agricultural sector plays an important role in the formation of the economic fabric and of which approximately 3100 km^2^ represents an irrigated area. The semiarid continental climate of the Haouz plain is characterized by an average annual rainfall of 250 mm and high temperature in summer (37.7°C on average maximum) and low in winter (4.9°C as the average of the minimum) [16]. The main agricultural production in the region remains cereal with nearly 5.6 million quintals in 2011–2012, or an average yield of 6.2 quintals per hectare [17].

**Figure 1:**
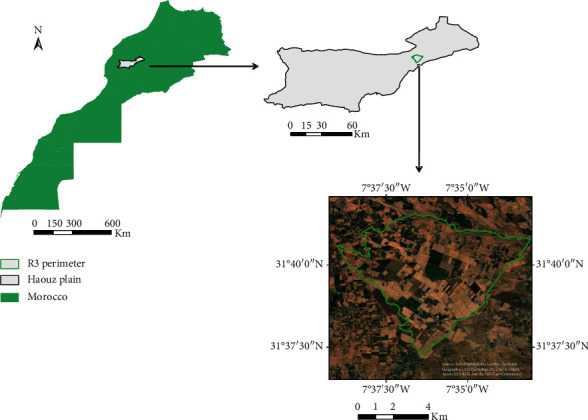
Test site location.

In this work, we are interested in the left part of the irrigated sectors, in the Haouz plain, called R3 perimeter and located in the Sidi Rahal region about 40 km east of the city of Marrakech (Figure 1). It is a sector irrigated by a gravity. It is divided into plots (several plots form blocks) of different size. The majority of plots are used for the production of cereal (46% of the areas surveyed in 2012), followed by tree crops (mainly olive and orange) and market gardening. A significant part, a quarter to a year depending on the year, is left fallow or not cultivated. The cereal is sown between November and January, reaches its maximum development in late March, and is harvested in early summer. The characteristics of this site (absent relief, regular, and large plot) make it a privileged study area for evaluating the contribution of satellite data in the extraction of information on changes in classes. For this, the R3 site has been intensively investigated in recent years (and still is) [5, 16, 18].

**Figure 2:**
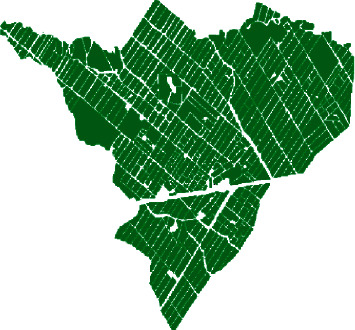
Division of R3 perimeter into 506 plots.

The different crops of the perimeter R3 were digitalized and subdivided into plots using ArcGIS software. These plots were created in such way to delineate each crop separately in order to identify the spectral responses and radar signal responses within the same plot. A total of 506 plots were recorded, with areas varying from 0.05 up to 5 hectares. We used very high spatial resolution images, updated for the year 2018 and provided by Google Earth Archives.  Figure 2 shows the division of the area into 506 plots.

### 2.2. In Situ Data

**Figure 3:**
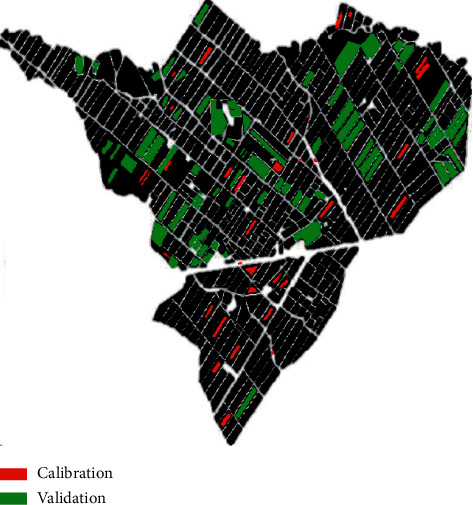
Spatial distribution of reference data over R3 perimeter.

Field data is used to extract profiles, calibrate or train classification algorithms, and validate the results (accuracy assessment of classified image). Samples were extracted during a field campaign carried out on April 2018 and were supplemented by samples extracted using the archives of high spatial resolution images (Spot) on Google Earth platform. The investigated plots are accompanied by detailed description providing information on the type of land cover and are divided into two groups, namely, calibration samples and validation samples.  Figure 3 illustrates the spatial distribution of the surveyed plots (calibration and validation). The land cover types were grouped into six main classes which were chosen in terms of abundance in the study area. This typology contains orange trees, olive trees, cereal, double cropping (cereal in winter + summer crops), fallow, and bare soil.

### 2.3. Remotely Sensed Data

The optical and SAR imagery were used in this work acquired from the two sensors Sentinel-2 and Sentinel-1, respectively. The choice of these two sensors is mainly due to the availability and cost of these products, as well as the high spatial, spectral, and temporal resolutions they offer.

#### 2.3.1. Sentinel-2 Images

ESA (https://sentinel.esa.int/web/sentinel/home) produces and distributes ortho-rectified Sentinel-2 data expressed in reflectance at the top of the atmosphere, the 1C level. Theia [19] produces and distributes level 2A data, corrected for atmospheric effects using the MAJA software developed thanks to the coordination between CNES and CESBIO [20–22]. This processing chain uses multitemporal information to detect clouds and their shadows, estimate the optical thickness of aerosols and the amount of water vapour, and correct for atmospheric effects. The data are freely downloadable from Theia official website, http://www.theia-land.fr/. The MAJA chain is an atmospheric correction and cloud detection system. It can accommodate time series of high-resolution images taken at constant or nearly constant viewing angles. It can process data from Landsat and Sentinel-2 satellites.

*(1) Data Processing.* S2 images were processed to derive products based on a vegetation index known as NDVI (Difference Normalized Vegetation Index). This index, presented by Tucker in 1979 [23], is now the most widely used vegetation index in remote sensing and indicates the importance and the dominance of vegetation on remotely sensed image. The transformation of the images into NDVI was carried out for the whole 20 images, in order to extract the phenological evolution of the classes, since it can be modelled by the NDVI profiles. NDVI is calculated from the normalized difference of the near infrared (NIR) and red (*R*) bands of S2 images according to the following formula:

(1) $$\begin{array}{c} \text{NDVI}=\;\frac{\text{NIR}-R}{\text{NIR}+R}. \end{array}$$

*(2) Acquired Scenes.* S2 images have been used for the present study. All available atmospheric corrected and cloudless S2 images, from January 1 to December 31 of 2018, have been downloaded from Theia Land Service website. These images resulted in 20 images covering the whole year of 2018 and well distributed over the 4 seasons (around 2 images per month), used to monitor the phenological evolution of the different classes chosen for the R3 test site.

#### 2.3.2. Sentinel-1 Images

Microwave signals are emitted by an antenna towards a particular region of the Earth surface for synthetic aperture radar (SAR) imaging. The microwave energy reflected back to the spacecraft is measured. SAR images are created using the radar concept, which takes advantage of the time delay of backscattered signals to create an image. The intensity of each pixel in a SAR image reflects the proportion of microwave backscattered from that ground region. The backscattering coefficient is a physical quantity that ranges from +5 dB for very light objects to −40 dB for very dark surfaces, with values ranging from +5 dB for very bright objects to −40 dB for very dark surfaces.

Sentinel-1 is a synthetic aperture radar (SAR) mission that provides continuous all-weather, day-and-night images at C-band in four imaging modes (EW, IW, SM, WV) with various spatial resolutions (10, 20, 60 m) and coverages. This mission is based on a constellation of two identical satellites, Sentinel-1A and Sentinel-1B, launched separately.

#### 2.3.3. Data Processing

*(1) Speckle Noise Removal Filtering*. Unlike optical imagery, SAR data is formed by coherent interaction of the transmitted microwave with the targets. Hence, it is affected by the speckle noise which arises from coherent summation of the signals scattered from ground scatterers distributed randomly within the pixels. A SAR image appears visually more noisy than an optical one. Therefore, a speckle noise removal filter is necessary before display and further analysis.

To reduce the speckle noise in the acquired S1 images, Enhanced Lee Filter [24] was applied. In the literature, there is a large family of filters [25–27]; therefore, Enhanced Lee Filter is one of the efficient filters used for SAR imagery in particular for areas with high degree of heterogeneity [28]. This filter is widely used for remote sensing applications [29, 30]. Enhanced Lee Filter, implemented in ENVI software used for crop classification, was applied in the different windows given by the software and the best speckle noise filtering was achieved in window of 5*∗*5 [31, 32].

To interpret and analyze the SAR imagery, another processing was applied to get the backscattering coefficient values. Therefore, the filtered images were transformed into decibels (dB). This transformation was done through the following formula:

(2) $$\begin{array}{c} X=10\;\log_{10}\;x, \end{array}$$

where *x* is the value of each pixel.

*(2) Texture Features*. Texture is a native spatial attribute of an image. Due to the sensitivity of the SAR backscatter to the homogeneity, orientation, spatial relationship, and type of ground objects, this kind of imagery represents certain texture features [33]. Therefore, the texture analysis is highly important while using SAR data.

Texture analysis is based mainly on the computation of textures features from an image. These features are determined from the statistical distribution properties of the image spectral tone for a certain neighborhood [34, 35]. Texture analysis statistics are classified into first-order, second-order, and higher-order statistics. The Gray Level Co-occurrence Matrix (GLCM) is one of the methods to calculate the second-order statistical texture features.

Texture analysis from GLCM offers crucial and reliable information on the spatial relationship of the pixels of an image [36]. In general, GLCM estimates the probability that pixel values (in moving windows) occur in a given direction and at a certain distance in the image [37]. There are many texture features computed by GLCM [38], three of which were calculated in this study, namely, the mean, variance, and correlation (Table 1).

The three S2 derived GLCM features were computed with a moving window size of 5 × 5, in all directions, based on the method [38]. These GLCM statistics were applied for both VV parallel polarization and VH cross polarization using the Sentinel toolbox of the software called SNAP (SeNtinel Application Platform).

*P*_*i*,*j*_ is a normalized gray tone spatial dependence matrix such that∑_*i*,*j*=0_^*N*−1^*iP*_*i*,*j*_=1; *i* and *j*, respectively, represent the rows and the columns for the mean, variance, and correlation measures; *μ* is the mean for the variance texture measure; and N is the number of distinct gray levels in the quantified image; (*μ*_*x*_, *μ*_*y*_), and (*σ*_*x*_,  *σ*_*y*_) are the means and standard deviations of *P*_*x*_ and *P*_*y*_, respectively, for the correlation texture measure [39].

*(3) Acquired Scenes.* An automatic processing chain generates single-date products and “ready-to-use” time series for a very large number of applications. Sentinel-1 data are ortho-rectified on the Sentinel-2 grid to facilitate the joint use of both missions. This product, named “S1Tiling,” has been developed within the CNES radar service, in collaboration with CESBIO, to generate calibrated, ortho-rectified and soon to be filtered Sentinel-1 time series images over any terrestrial region of the Earth. It is based on the ortho-rectification application of radar images from the Orfeo Tool Box. The images obtained are superimposable on the Sentinel-2 optical images, as they use the same geographical reference frame. We can therefore have access to Sentinel-1 data acquired on Sentinel-2 tiles.

**Figure 4:**
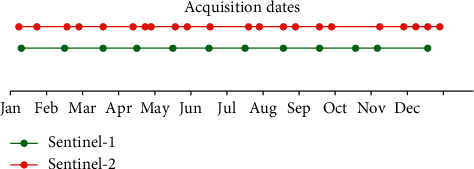
Distribution of acquisition dates of SAR and optical images.

One SAR image per month was downloaded in accordance with the important dates in terms of vegetative cycles of the crops over the study area. 12 SAR images were acquired for this study. These images have been chosen in such way that they are acquired no more than 2 days before or after the date of acquisition of the optical image of the same period.  Figure 4 shows the temporal distribution of the acquisition dates of the downloaded S2 and S1 images.

## 3. Methodology

The methodological approach used in this work consists of 4 main steps:

1. Data acquisition
2. Data preparation and preprocessing
3. Extraction and analysis of profiles (NDVI, VV, VH, VV/VH) and classification
4. Evaluation of obtained results by the confusion matrix (OA and K)

**Figure 5:**
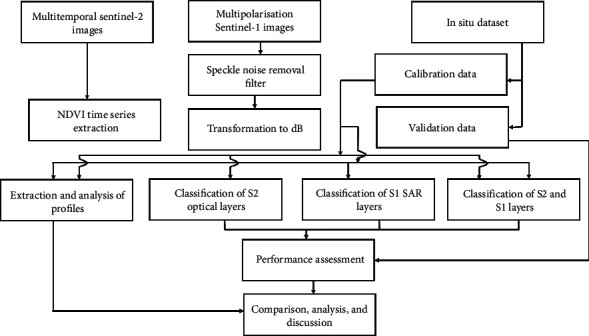
Methodological approach applied in the study.

Figure 5 illustrates the sequence of the different steps just mentioned. Since we have already presented the first two steps in the previous sections, we will subsequently detail the 3rd and 4th steps.

### 3.1. Temporal Optical NDVI and SAR Backscatter Profiles Extraction

**Figure 6:**
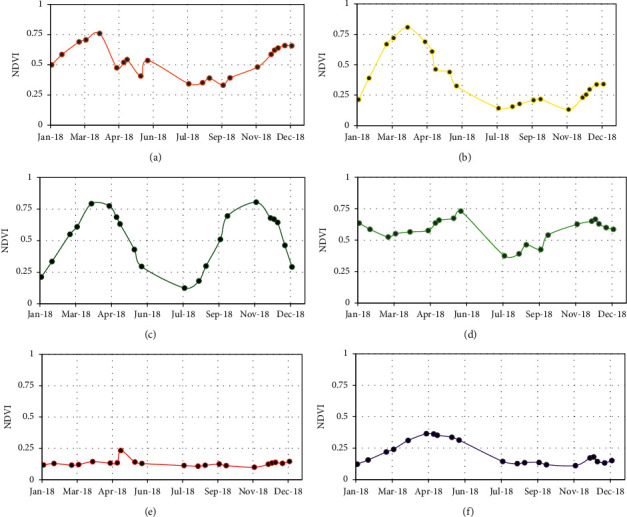
NDVI profiles of the different classes chosen in zone R3 for the year 2018. (a) Oranges. (b) Cereal. (c) Double cropping. (d) Olive. (e) Bare soil. (d) Fallow.

**Figure 7:**
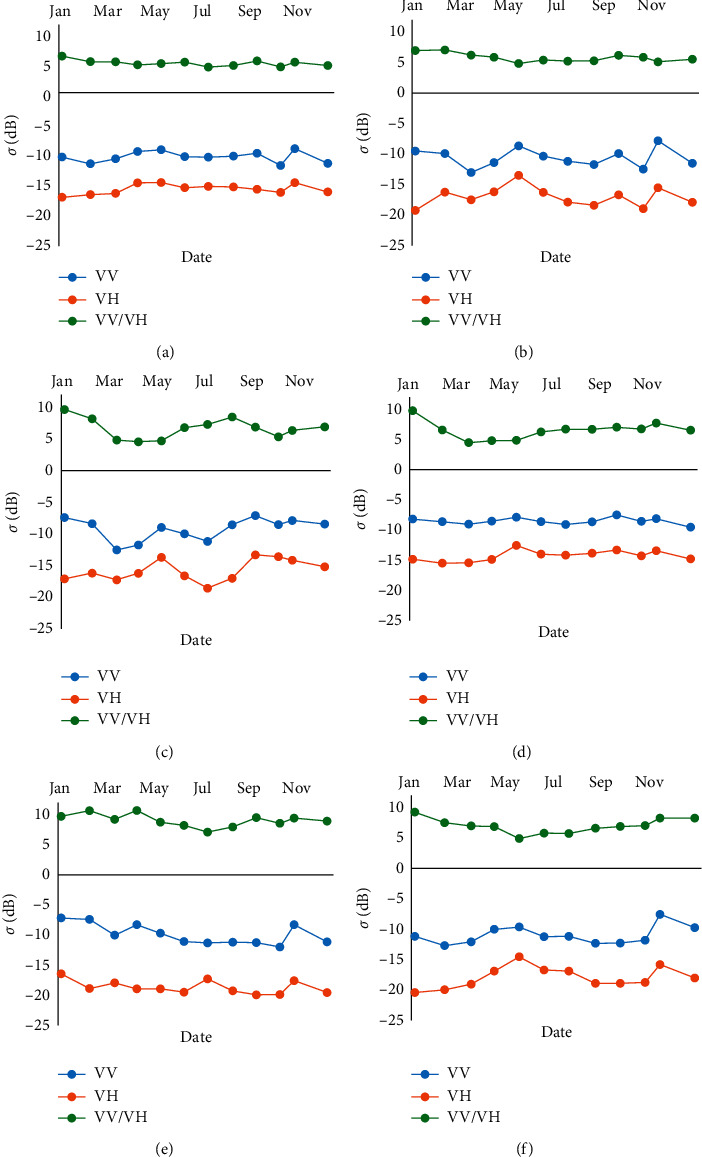
VV, VH, VV/VH, and VV-VH profiles for the different classes chosen in zone R3 for the year 2018. (a) Oranges. (b) Cereal. (c) Double cropping. (d) Olive. (e) Bare soil. (d) Fallow.

For each of the chosen classes, the NDVI, VV, VH, and VV/VH profiles were created, in order to study the possible confusions or separability to be expected between these different classes, and to be able to analyze and interpret the classification results. The temporal evolution of NDVI profiles allows the modelling of the dynamics of land cover types, and particularly the phenological evolution of the crop-type classes, which makes them relatively interpretable. On the other hand, the radar profiles are more complex for the vegetative classes, reflecting the evolution of the proportion of the backscattered signal, mainly influenced by the surface roughness of the canopy as well as its water content. Figures 6 and 7 show, respectively, for each of the chosen classes, the temporal evolution of NDVI, and that of the backscattered signals in the C-band (in VV, VH, VV/VH polarizations).

### 3.2. Machine Learning-Based Classification

The classification was performed using three supervised machine learning algorithms implemented in ENVI, namely, maximum likelihood (ML), support vector machine (SVM), and neural network (ANN).

 
*ML Classifier.* It is a supervised classification technique that relies on the statistics of class signatures extracted directly from the satellite imagery or of training samples representing different land cover types chosen on the basis of the ground truth data collected during field campaigns [5, 40]. ML is a pixel-based algorithm that based on a multivariate probability density function of classes [41]. The likelihood of a pixel to be belonging to each of the considered classes is calculated. Then, this pixel is affected to a class having the highest probability of pixel belonging. MLC is a one of the most commonly used supervised classification methods in remote sensing to derive land use/cover maps [3, 42–44].
 
*SVM Classifier.* The support vector machine (SVM) is one of the most useful machine learning algorithms. It is based on statistical learning theory and has been extensively exploited in remote sensing for LC/LU mapping and crop classification [44]. The main advantage of SVM method is the ability to classify high dimensional data with small set of training samples [45]. SVM works with pixels in the boundary of considered classes which are named support vectors [46, 47]. For the complex data that cannot be separated using linear hyper-planes, optimal hyperplane separating the different classes by nonlinear mapping functions, called kernel functions, can be defined. Several kernel functions can be used with SVM classifier. However only four of them which are linear, polynomial, radial basis function (RBF), and sigmoid kernels have been commonly used to classify satellite data [48]. RBF kernel was used for the present study.
 
*ANN Classifier.* ANN classifier was originally created, as a mathematical model, for data analysis and pattern recognition in order to mimic the analytical operations and neural storage of the human brain. It is such parallel system of calculation which consists of large number of basic processors with interconnections [49]. ANN is extensively adopted machine learning technique for LC/LU mapping [44, 50, 51]. It has the ability to learn from training ground samples and store the pattern of the each input variable (land cover classes in the present study). After the training step, new data (pixels of satellite image) is introduced to ANN classifier; it recognizes the pattern from this data and classify it.

In the present study, we have restricted ourselves to the use of supervised methods since these techniques generally provide better results in the production of LC/LU maps. First, we started by classifying the time series of NDVI, VV, VH, and VV/VH. We then combined the data from the two sensors to explore different possible scenarios.  Table 2 describes these scenarios in the order followed in the classification step.

### 3.3. Accuracy Assessment

The evaluation of the classification results can be done qualitatively by comparing the images or quantitatively by measuring the accuracy of the LC/LU classification using statistical tools such as the confusion matrix and the Kappa index [52, 53]. Confusion matrices were calculated to reveal not only the general errors made at the level of each class when interpreting the results, but also errors due to confusion between LC/LU classes [54, 55].

The overall accuracy (OA) of the classification, used as one of evaluation metrics, is given by the average of the percentages of correctly classified pixels in the following equation:

(3) $$\begin{array}{c} \text{OA}=\frac{\sum_{i=1}^{k}X_{ii}}{N}, \end{array}$$

where *X*_*ii*_ is the number of diagonal pixels (correctly classified) and *N* is the total number of pixels.

The second assessment metric is the Kappa coefficient (*K*), which is calculated by the formula of the following equation:

(4) $$\begin{array}{c} K=\frac{A_{0}-\;A_{c}}{1-\;A_{c}} \end{array}$$

where *A*_0_ is the obtained OA or actual percentage of classified land is covers and *A*_*c*_ is the probability of obtaining a correct classification.

### 3.4. Postclassification

After the classification process, the classified images including SAR bands showed a law sharpness. A majority filter therefore was applied to get smoothed images. It is a logical filter applied to a classified image. In this process, the number of pixels assigned to each of the classes is calculated and if the center's pixel is not a member of the majority class (including 3, 5 or more pixels according to the considered window), it is given the label to the majority class. Such filters are used in order to smooth the classified images by the number of pixels allocated to each of the classes counted and, if the center pixel is not a member of the majority class (containing five or more pixels within the window), it is given the label of the majority class. The effect of this algorithm is to smooth the classified image by weeding out isolated pixels, which were initially given labels that were dissimilar to the labels assigned to the surrounding pixels [56].

## 4. Results and Discussion

### 4.1. Temporal Analysis of the Profiles

#### 4.1.1. NDVI Profiles

The NDVI vegetation index provides information on the importance or the dominance of the vegetation cover; it allows the modelling of the phenological stages of the different crops. For annual crops, for example, the start of the season begins when the rate of increase in NDVI values is greater than the previous successive observations during the period of vegetation growth. The end of the season is defined as the time during the maturation period when there is a significant decrease in NDVI. Generally, it corresponds to the period during which chlorophyll activity gradually decreases [27]. These evolutions can be seen in Figure 6 graphs B, C, and F for cereal, double cropping, and fallow. The NDVI spectral profiles of these crops reflect their seasonality. The amplitude of the graphs becomes important during periods of vegetation development.

Wheat and barley (grouped in the cereal class because of their high phenological similarity) are grown in early December, reach their maximum development at the end of March, and are harvested in mid-May and no later than early June (graph B). The same description remains valid for the fallow class (graph F), because this natural vegetation benefits from the presence of winter rains and grows over the same period of March. For the time profile of the double cropping class (graph C), it is the union of the profiles of two crops: cereal and vegetable crops or corn.

Orange and olive belong to perennial crops group, yet we have separated them. The NDVI value of these classes is generally over 0.3 throughout the year (Figure 6, graphs A and D). Decreases in NDVI values of tree crops in summer are due to water stress resulting from water scarcity and increased temperatures over this period and in addition sometimes due to leaf loss. The bare soil class is relatively easy to detect due to the absence of vegetation resulting in NDVI values that not exceeding 0.20 (Figure 6, graphs E).

#### 4.1.2. VV, VH, and VV/VH Profiles

The profiles in Figure 7 illustrate the temporal evolution in intensity (SAR backscattering coefficient expressed in decibels of the VV, VH, and VV/VH polarizations), of the 6 selected covers, orange, olive, cereal, double cropping, fallow, and bare soil. Unlike NDVI profiles, radar profiles are more complicated to interpret.

The first thing noticed is that, for all the studied classes, the values of intensity in parallel polarization VV (values between −7 dB and −12 dB) are above those in cross-polarization VH (values between −12 dB and −20 dB), while the intensity values of the VV/VH ratio are positive (varying between 4 dB and 10 dB). In particular, for the double cropping class, the VH and VV/VH signals show the most marked seasonality over the entire period with amplitudes of the order of 3 dB and 5 dB, respectively, linked more in VH polarization to the periods of leaf activity in phase with NDVI. This may be at the origin of the distinction of the dual culture class from the others.

For the tree crops, oranges class showed “Presque” a stable value of VV/VH signal with slight variations, mainly in May, August, and November. Similar variation of amplitudes in the same period was recorded also for VV and VH signals (varying between min of −11 and a max of −8 dB for VV signal, and between min of −16 and max of −14 for VH signal). Compared to olive NDVI profile, the period of variation of SAR backscatter signals is the same months when the NDVI values decrease. This shift can be explained by the increase of temperature which is related directly to soil moisture. The second crop tree, oranges class, presented significant decrease, of VV/VH ratio values, during the period between February and May (varying between min of 4 dB and max of 9 dB), while VV and VH signals showed negligible shifts.

For cereal (barley and wheat), barley and wheat are more redundant annual crops in the study area. Both of these crops have similar phenology and plant structure; we therefore considered them as same crop class, namely, cereal. For this class, the VV and VH signals represented same variations as double cropping especially during spring and summer (from January to July). Those shifts can reflect the high leaf activity of cereal during this period compared with NDVI values. During the rest of the year, some variations were observed in particular November and December due to precipitations. For cereal's VV/VH signal, no significant shifts were recorded. Finally, unlike the Fallow's and bare soil's NDVI profiles, the backscatter signals' values are distinguished.  Table 3 summarizes the ranges and amplitudes of signals for all classes over all months of the year.

### 4.2. LC/LU Classification Results

This section is about the performance assessment of the classification algorithms, applied to the crop-type discrimination in *R*3 perimeter, from the time series of both Sentinel-1 and Sentinel-2 data. Quantitative results, using the performance metrics (OA, *K*), were presented and followed by qualitative results (visually analyzing the overall quality of classified images).

#### 4.2.1. Quantitative Results

First of all, the results corresponding to the initial scenarios of noncombined Sentinel products are presented and followed by the classified images for these scenarios. Then, we studied the classification results of the combined products of S1 and S2 data. Finally, we analyzed these results by highlighting the scenarios leading to better OA and K indices. The results of the first group (noncombined products) are grouped together in Table 4.

The results of the product classifications from each of the two S1 and S2 sensors confirm the superiority of the optical data and the SVM algorithm in terms of LC/LU classification and the performance, respectively, especially that we had problems of overestimation with the ML and ANN algorithms, even if the latter is one of the most powerful machine learning classifiers. The resulting OA of the NDVI time series classification is 85.83% (*K* = 0.81), while the best OA values were obtained by classifying the time series of 2 scenarios (VV and VH) and (VV, VH, and VV/VH). These OA are equal to 75.43% (*K* = 0.67) and 75.42% (*K* = 0.67), respectively. We noticed that the addition of the VV/VH band provided no improvement to VV and VH, while the inclusion of textural characteristics improved the classification up to 76.72% (*K* = 0.68).

Taking into consideration that SAR sensors are not primarily intended for LC/LU mapping, the classification of radar products presents acceptable results despite being inferior to those obtained by NDVI (optical data). The Kappa index being between 0.61 and 0.80 indicates “strong agreement” according to Landis and Koch, 1977 [28]. The results of the second group (combined optical and SAR products) are presented in Table 5.

The classification results of the S1 and S2 combined products show a slight increase (to a maximum of approximately 2%) in the specifications of scenarios 12 (NDVI; VV), 13 (NDVI; VH), and 14 (NDVI; VH; texture). On the other hand, there is a slight decrease for the rest with the same rate. As for the first group, some scenarios were overestimated by the ML and ANN machine learning algorithms.

#### 4.2.2. Qualitative Results

**Figure 8:**
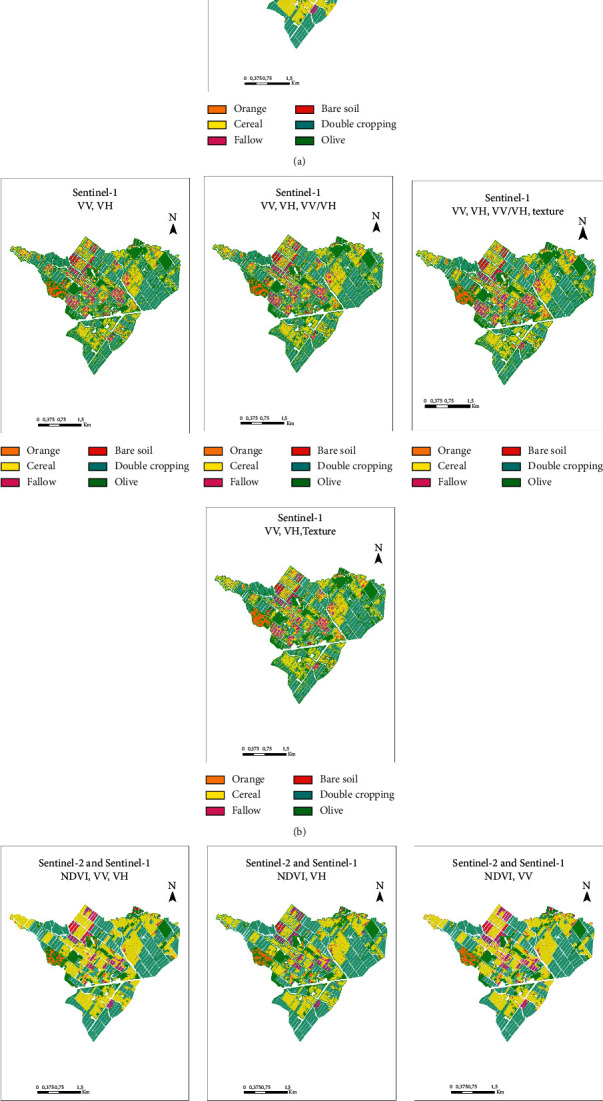
Classified images resulting from the best classification accuracies.

In order to visually compare the quality of the classified images, we then presented in Figure 8 the images obtained from the scenarios that resulted with higher accuracy. We noticed some differences between the classifications of S1 and S2 products. In fact, first of all we observed inconsistencies in classifications with the olive tree and fallow classes. Moreover, the quality of classified SAR images is lower than those classified with NDVI images in terms of sharpness even though the effect of speckle was reduced after applying the noise removal filter. The combined products resulted in very sharp images, which is due to the complementary contribution of optical data. The presence of isolated pixels on homogeneous plots over the entire area contributed to the “noisy” appearance of classified SAR products. Therefore, we have tried to improve the quality of these images and the accuracy of the results by applying post-processing which consists of smoothing classifications containing SAR products.

#### 4.2.3. Postclassification: Smoothing

**Figure 9:**
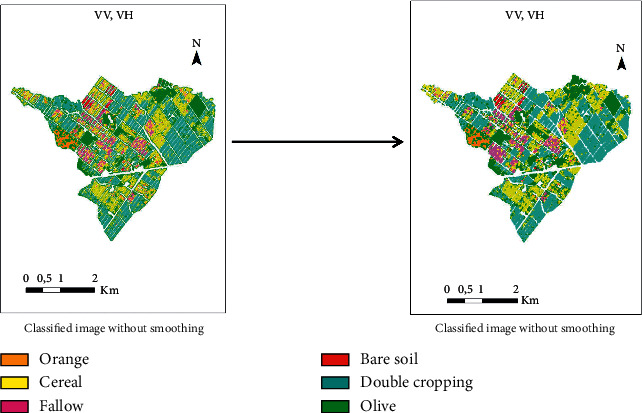
Improved quality of one of the classified images from radar products after smoothing.

When the classification of scenarios containing SAR products is carried out, we notice in the images a lack of sharpness in the definition of the classified plots; the images seem “noisy.” We then apply a smoothing on these images, through the command of ENVI called “Majority/Minority Analysis.” This command filtered the image by replacing the value of the central pixel of a window of size *n* × *n* (where we must define n) by the majority value located in this window. In order to avoid an “oversmoothing,” we have chosen a window of size 3 × 3. We present in Figure 9 an example of a classified SAR image before and after smoothing. Visually, the quality has improved, and this product, which is derived from S1 data only, looks more like the classified image from the NDVI time series.

This process was applied to the scenarios containing SAR products and which gave the best results.  Table 6 displays the overall accuracies and the Kappa indices of these scenarios before and after applying the smoothing.

**Figure 10:**
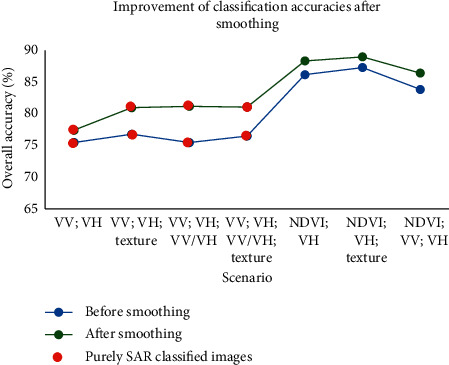
Increased precision of classified images containing radar products that gave the best precision.

In order to better visualize these improvements, Figure 10 shows the evolution and the increase in the values of the OAs after smoothing.

**Figure 11:**
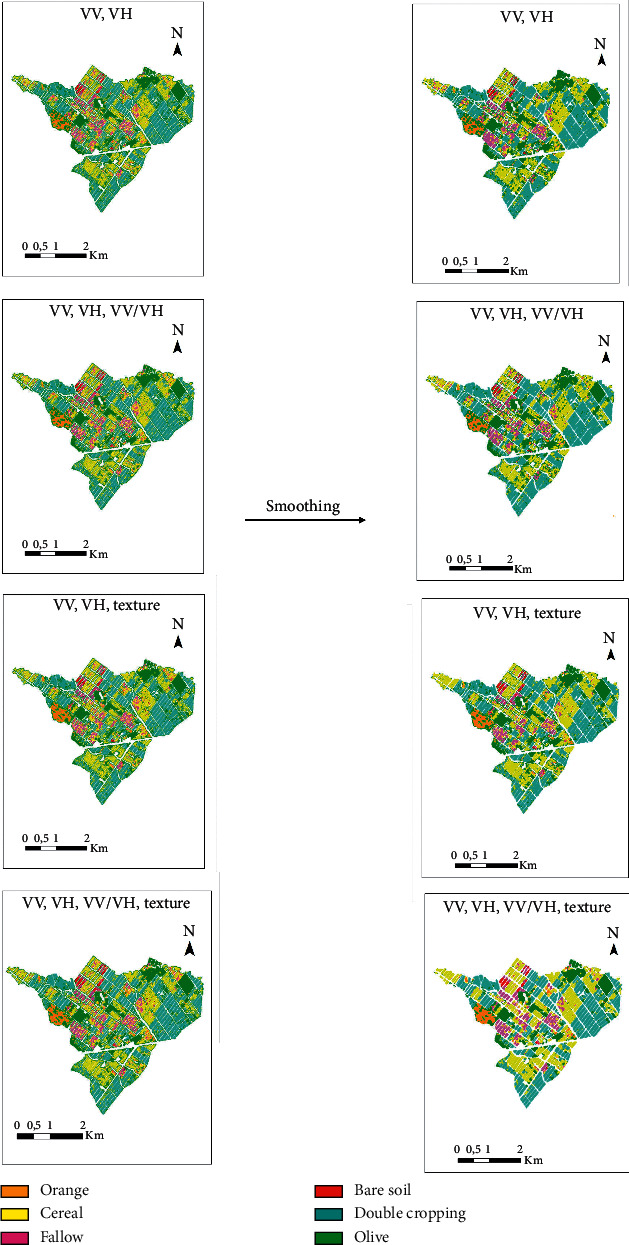
Smoothing of 4 SAR classification scenarios (VV; VH), (VV; VH; texture), (VV; VH; VV/VH), and (VV; VH; VV/VH; texture).

Figure 11 illustrates smoothed images derived from purely SAR products of the scenarios in Table 6.

**Figure 12:**
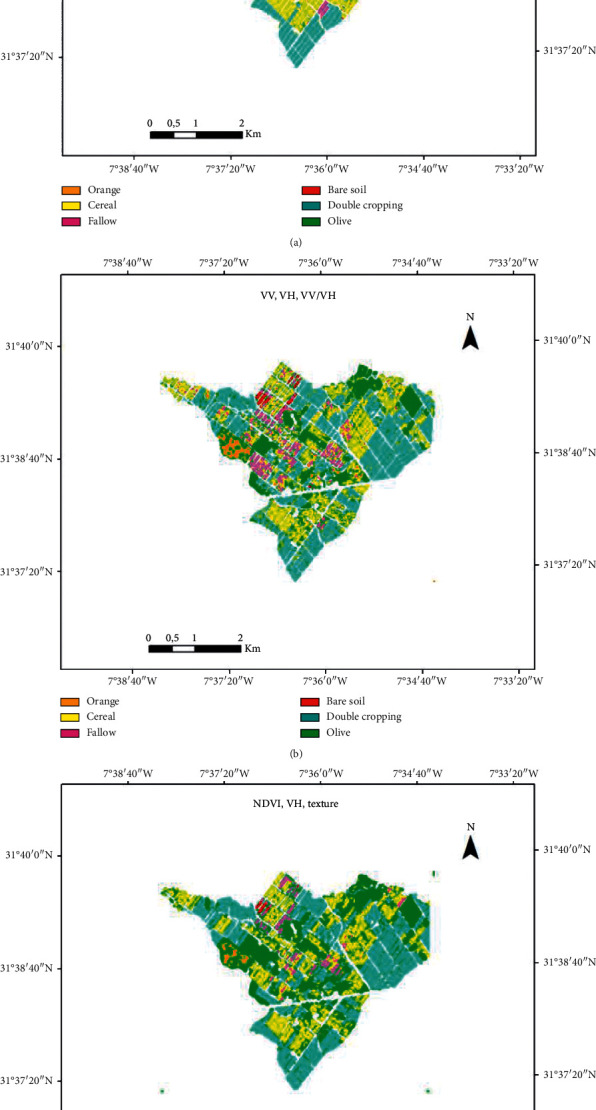
Final land use maps. (a) Optical (85.83%, *K* = 0.81), (b) radar (81.12%, *K* = 0.75) and (c) optical combined with SAR (88.90%, *K* = 0.85).

Figure 12 illustrates the best three thematic products series, namely, (1) crop-type map from NDVI time series, (2) crop-type map from SAR time series (VV, VH, VV/VH, and texture) smoothed, and (3) crop-type map from combined optical and SAR data (NDVI, VH, and texture).

## 5. Discussion

In general, NDVI profiles model the phenological behavior of classes, and compared to VV, VH, and VV/VH profiles, they showed great class separability which facilitates their discrimination when applying classification algorithms. By quantifying the results using confusion matrices, the separability was translated by the deviation found on the overall accuracies and the Kappa indices, with around 85% (*K* = 0.81) and around 77% (*K* = 0.69) of OA on the classifications of NDVI (optical data) and a combined product of VV, VH, and texture (best case scenario of the purely SAR data). Product integration of the two sensors S1 and S2 slightly increased accuracy compared to using data from S2 only by about 2% (maximum).

A visual interpretation of the quality of the results showed that classifications of multiband images containing S1 data are still “noisy” despite these images having been filtered before being classified. In order to further improve the classification performance, we used a post-classified product smoothing technique. Effectively, the results improved and the classification of data derived from S1 only reached a remarkable OA of 81.12% (*K* = 0.75) for the best scenario (VV, VH, and VV/VH). The smoothing also contributed to the improvement of the classification accuracy of combined products (S1 and S2), with an increase of approximately 3% to reach the best accuracy over the 20 scenarios covered in total, which is worth about 89% of OA (with *K* = 0.85).

## 6. Conclusions

LC/LU mapping is, for land management, a necessary tool for understanding, analyzing, and monitoring land cover dynamics in order to better exploit land. In addition, the availability of high spatio-temporal resolution satellite data, which have the advantage of covering surfaces at all scales (local, regional, and continental), offers the possibility of carrying out this mapping. The use of optical remote sensing including radiometric indices, combined with the textural characteristics of SAR remote sensing imagery, is generally accepted as a mean of improving classification performance [39, 57–61]. The objective of this study was to identify and map the different crop types in the irrigated R3 perimeter using high-resolution multidate satellite images S1 and S2 of the year 2018. The R3 sector characterized by a semiarid climate is located around the city of Sidi-Rehhal about 40 km southeast of the city of Marrakech.

The classification of the NDVI time series alone resulted in an OA of about 86% (*K* = 0.81), while the best result was found by the integration of the S1 and S2 products, and particularly by using NDVI combined with the VH cross-polarization and textural characteristics (about OA = 87%, *K* = 0.82 before smoothing and OA = 89%, *K* = 0.85 after smoothing). We noticed that, in general, the parallel VV polarization improves accuracy very slightly, while the derived VV/VH band hardly influences the quality of the classifications. The integration of all S1 products (VV, VH, VV/VH, textural characteristics) with the NDVI series resulted in decreased OA and K coefficient. The worst result was found for the S1 product classification only, with an OA not exceeding, for the best case scenario, 77% (*K* = 0.65). A visual interpretation of the quality of the results showed that classifications of combined images containing SAR data are still “noisy” despite these images having been filtered before being classified. In order to further improve the accuracy, we used a post-classified product smoothing technique. Effectively, the results improved and the classification of data derived from S1 only reached a significant OA of 81.12% (*K* = 75) for the best scenario (VV, VH, and VV/VH). The smoothing also contributed to the improvement of the classification accuracy of combined products (S1 and S2), with an increase of approximately 3% to reach the best OA on a total of 63 classifications, which is worth about 89% (*K* = 0.85).

The results are largely in agreement with the literature. For our best classifications, it can be said on the one hand that the integration of S1 and S2 has increased the accuracy and quality of classification compared to the single use of S1 or S2. On the other hand, the results found by using S1 products only hold promise for the use of radar data for ground mapping as an alternative to optical data.

As perspective, it will be necessary to test the synergistic use of S1 and S2 sensors for mapping a larger area (e.g., the Haouz plain). We can then expect a decrease in performance, linked to confusion due to the dimensionality and diversity of the land cover types. To overcome this, we can consider expanding the database, using very high resolution image processing techniques for the detection of orange and olive trees as well as in-depth work on the smoothing and parameterization of profiles.

## Figures and Tables

**Table 1:** Mathematical formulas for calculating texture features using GLCM statistics: the mean, variance, and correlation.

Texture	Formula
Mean	∑_*i*,*j*=0_^*N*−1^*iP*_*i*,*j*_
Variance	∑_*i*,*j*=0_^*N*−1^*iP*_*i*,*j*_(*i* − *μ*)²
Correlation	(∑_*i*,*j*=0_^*N*−1^*iP*_*i*,*j*_ − *μ*_*x*_*μ*_*y*_)/*σ*_*x*_*σ*_*y*_

**Table 2:** Different scenarios (noncombined and combined) from multisensor bands to classify.

Scenario	Layers	Layers used for classification	Sensor
1	Noncombined (single sensor)	NDVI	S2
2		VV	S1
3		VH	S1
4		VV/VH	S1
5		Texture	S1
6		VV; VH	S1
7		VV; VH; texture	S1
8		VV; VV/VH	S1
9		VH; VV/VH	S1
10		VV; VH; VV/VH	S1
11		VV; VH; VV/VH; texture	S1

12	Combined	NDVI; VV	S1 and S2
13		NDVI; VH	S1 and S2
14		NDVI; VH; texture	S1 and S2
15		NDVI; VV/VH	S1 and S2
16		NDVI; VV; VH	S1 and S2

17	(multisensor fusion)	NDVI; VV; VV/VH	S1 and S2
18		NDVI; VH; VV/VH	S1 and S2
19		NDVI; VH; VV/VH; texture	S1 and S2
20		NDVI; VV; VH; VV/VH	S1 and S2

**Table 3:** Minimum values, maximum values, and amplitudes of the VV, VH, and VV/VH signals for the different classes.

Classes	Signal VV			Signal VH			Signal VV/VH		
	Min	Max	Amplitude	Min	Max	Amplitude	Min	Max	Amplitude
Orange	−11.5	−8.7	2.8	−16.9	−14.4	2.5	4.9	6.7	1.8
Olive	−9.5	−7.5	2.1	−15.5	−12.5	3.0	4.5	9.7	5.3
Cereal	−13.0	−7.8	5.2	−19.2	−13.5	5.7	4.9	7.1	2.2
Double cropping	−12.5	−7.1	5.4	−18.6	−13.3	5.3	4.6	9.7	5.1
Fallow	−12.7	−7.6	5.2	−20.4	−14.5	5.9	4.9	9.2	4.3
Bare soil	−12.0	−7.2	4.8	−19.6	−16.4	3.5	7.1	10.7	3.6

**Table 4:** Overall accuracies and Kappa coefficients of noncombined scenarios.

	SVM		ML		ANN	
	OA	*K*	OA	*K*	OA	*K*
NDVI	85.83	0.81	84.38	0.79	81.54	0,76
VV	64.81	0.52	62.56	0.50	60.45	0,48
VH	71.35	0.61	56.96	0.44	66.90	0,55
VV/VH	51.28	0.33	45.98	0.29	44.86	0,27
Texture	58.22	0.44	50.92	0.38	54.85	0,40
VV; VH	75.43	0.67	71.15	0.60	65.62	0,56
VV; VH; texture	76.72	0.69	72.83	0.63	54.89	0,41
VV; VV/VH	72.97	0.59	66.96	0.55	58.38	0,38
VH; VV/VH	72.10	0.62	66.95	0.55	Overestimation	
VV; VH; VV/VH	75.42	0.67	Overestimation		Overestimation	
VV; VH; VV/VH; texture	76.45	0.68	Overestimation		55.69	0.42

**Table 5:** Overall accuracy and Kappa indices of combined scenarios.

	SVM		ML		ANN	
	OA	*K*	OA	*K*	OA	*K*
NDVI; VV	82.93	0.77	81.24	0.74	59.98	0.45
NDVI; VH	86.10	0.81	84.46	0.78	72.18	0.62
NDVI; VH; texture	87.22	0.82	85.68	0.80	51.89	0.38
NDVI; VV/VH	82.33	0.80	81.00	0.74	58.11	0.43
NDVI; VV; VH	83.78	0.78	82.83	0.76	Overestimation	
NDVI; VV; VV/VH	83.37	0.77	83.39	0.76	Overestimation	
NDVI; VH; VV/VH	84.60	0.79	82.39	0.75	Overestimation	
NDVI; VH; VV/VH; texture	85.52	0.80	84.36	0.78	55.09	0.42
NDVI; VV; VH; VV/VH	83.83	0.78	Overestimation		Overestimation	
NDVI; VV; VH; VV/VH; texture	85.20	0.80	Overestimation		55.59	0.42

**Table 6:** Improved overall precision and Kappa indices by applying smoothing.

	Before smoothing		After smoothing	
	OA	*K*	OA	*K*
VV; VH	75.43	0.67	77.36	0.67
VV; VH; texture	76.72	0.69	80.91	0.74
VV; VH; VV/VH	75.42	0.67	81.12	0.75
VV; VH; VV/VH; texture	76.45	0.68	81.01	0.75
NDVI; VH	86.10	0.81	88.26	0.84
NDVI; VH; texture	87.22	0.82	88.90	0.85
NDVI; VV; VH	83.78	0.78	86.35	0.81

## Data Availability

Sentinel‐1 and Sentinel‐2 were acquired over the study area and used for the present work. Sentinel‐2 images can be downloaded from the Theia CNES website: https://theia.cnes.fr/. Sentinel‐1 data can be downloaded from PEPS CNES website: https://peps.cnes.fr/. The SAR imagery was preprocessed using the Sentinel Application Platform (SNAP) and ENVI software
